# Estimation of Recurrence of Colorectal Adenomas with Dependent Censoring Using Weighted Logistic Regression

**DOI:** 10.1371/journal.pone.0025141

**Published:** 2011-10-31

**Authors:** Chiu-Hsieh Hsu, Yisheng Li, Qi Long, Qiuhong Zhao, Peter Lance

**Affiliations:** 1 Division of Epidemiology and Biostatistics, University of Arizona, Tucson, Arizona, United States of America; 2 University of Arizona Cancer Center, Tucson, Arizona, United States of America; 3 Department of Biostatistics, University of Texas MD Anderson Cancer Center, University of Texas, Houston, Texas, United States of America; 4 Department of Biostatistics and Bioinformatics, Emory University, Atlanta, Georgia, United States of America; Genentech Inc., United States of America

## Abstract

In colorectal polyp prevention trials, estimation of the rate of recurrence of adenomas at the end of the trial may be complicated by dependent censoring, that is, time to follow-up colonoscopy and dropout may be dependent on time to recurrence. Assuming that the auxiliary variables capture the dependence between recurrence and censoring times, we propose to fit two working models with the auxiliary variables as covariates to define risk groups and then extend an existing weighted logistic regression method for independent censoring to each risk group to accommodate potential dependent censoring. In a simulation study, we show that the proposed method results in both a gain in efficiency and reduction in bias for estimating the recurrence rate. We illustrate the methodology by analyzing a recurrent adenoma dataset from a colorectal polyp prevention trial.

## Introduction

The effect of a colorectal polyp prevention trial is often evaluated on recurrent adenomas as determined by colonoscopy at follow-up. The follow-up colonoscopy is usually scheduled at the end of the prevention trial (e.g. three years after start of the intervention). The corresponding recurrence status data are often analyzed using logistic regression. However, in a typical colorectal polyp prevention trial, a certain percentage of (often around 50%) participants have their follow-up colonoscopy before the end of the trial. If a participant drops out from the study at the time of an early follow-up colonoscopy without having any recurrent adenomas, this participant should be treated as right censored at the dropout time. Logistic regression that treats all participants censored before the end of the trial as having no recurrence at the end of the trial tends to underestimate the recurrence rate [1].

Based on the study design, each participant's recurrence is only known up to happen either before or after the follow-up colonoscopy. Hence, the recurrence status data can be considered as current status data, where the actual occurrence of an event is only known to the extent that it has or has not occurred before a single observation time. Survival analysis approaches for current status data, including the nonparametric maximum likelihood estimator (NPMLE) [2]–[4], inverse probability of censoring weighted (IPCW) estimators [5]–[8] and the semi-parametric regression methods [9]–[10], can then be used to estimate the recurrence rate to avoid the potential estimation bias due to inappropriate handling of the censored observations. However, none of those methods incorporate the possibility of cure into estimation. For a colorectal polyp prevention trial, the censoring rate is typically higher than 50% suggesting a possibility of cure. In addition, approximately 50% of the participants have their only follow-up colonoscopy at the end of trial and these data contribute little information to estimating a recurrence-free survival curve. These issues create difficulties in estimating the recurrence rate using the methods that do not incorporate the possibility of cure.

A different class of semi-parametric regression models has been proposed to handle current status data with a possibility of cure [11]–[12]. These semi-parametric regression models mainly focus on estimating the recurrence rate conditional on covariates. However, our main interest is in estimating the marginal recurrence rate through the use of covariates. In addition, those participants (approximately 50%) who have their only follow-up colonoscopy at the end of the trial similarly contribute little information to estimating the survival curve and will also create difficulties in estimating the recurrence rate for those models. Furthermore, those models also rely on a fully specified relationship between the covariates and the hazard rate, and the assumption of independent censoring of the time to recurrence. In contrast, we seek an approach focusing on estimating the recurrence rate at the end of the trial that does not require special handling of the possibility of cure and does not rely on the independent censoring assumption. We, therefore, expect such an approach to be more stable than existing survival analysis techniques.

Hsu et al. [1] proposed to directly estimate the recurrence rate at the end of the trial to avoid the problem associated with the possibility of cure in estimation. The next challenge was how to deal with censored observations. They adapted the idea in [13] to modify logistic regression using a weight function to account for participants' variable censoring time (i.e., follow-up colonoscopy time) for estimating the recurrence rate at the end of the trial. They treated a participant censored before the end of the trial differently from one censored at the end of the trial in the estimation of the recurrence rate. They showed that the weighted logistic regression method produces reasonable estimates even under a high prevalence rate of early follow-up colonoscopy. The performance of the weighted logistic regression method heavily depends on the assumption of independent censoring of the time to recurrence, and can produce biased estimates in the case of dependent censoring. Unfortunately, a participant's decision of having an early colonoscopy can be associated with his/her elevated risk of recurrence, e.g. based on the individual's family history of colorectal cancer. This thus induces dependent censoring into the recurrence time data. As a result, the weighted logistic regression method will produce a biased estimate of the recurrence rate.

In a colorectal polyp prevention trial, apart from the patients' recurrence status, additional variables potentially associated with the risk of recurrence (e.g. age and gender) are usually collected as well, which can be considered as auxiliary variables. These auxiliary variables can be used to define risk groups in each of which the participants have a similar risk of recurrence and, furthermore, induce approximate independent censoring within each risk group [14]. Specifically, we propose to fit two working proportional hazards (PH) models, one for the recurrence time and the other for the censoring time. In the model for the recurrence time, midpoint imputation is used to convert the interval censored recurrence time data to right censored data. In the model for the censoring time, observed censoring time data are used. We derive two risk scores (one from each PH model) to reduce the auxiliary variables into two scalars that are linear combinations of the auxiliary variables. We first show that if one of the two working PH models is correctly specified, censoring is independent of the risk of recurrence conditional on the two risk scores and then expect the approach to be robust to the model misspecification. We then propose to use these two risk scores to categorize participants into risk groups and then perform the weighted logistic regression [1] in those risk groups. If the auxiliary variables used to define the risk scores are predictive of recurrence, the analysis using the information from the auxiliary variables should be more efficient than that without using the information. In addition, if the auxiliary variables are also predictive of censoring, the analysis using the information from the auxiliary variables can reduce bias due to dependent censoring.

Our main interest in this paper is to estimate the treatment-specific recurrence rate at the end of the trial in the presence of multiple auxiliary variables that are predictive of recurrence and potentially time to colonoscopy (therefore potential dependent censoring). We are particularly interested in comparing the performance between the proposed method and the NPMLE method under a high rate of early follow-up colonoscopies. In addition, we are also interested in exploring the magnitude of the biases in the estimates of the recurrence rate derived from 1) the weighted logistic regression method ignoring the auxiliary variables, and 2) the simple sample proportion approach.

This paper is organized as follows. In Section 2, we review the weighted logistic regression method, and show the utility of the two derived risk scores for proposing an extended weighted logistic regression method that accounts for dependent censoring. In Section 3, we apply the models to a data set from a ursodeoxycholic acid colorectal adenoma prevention (UDCA) trial. In Section 4, we study the finite sample properties of the method through simulations. A discussion follows in Section 5. Some technical details are given in Appendix S1.

## Methods

Generically, let *X* denote the time to recurrence, *T* denote the time to censoring (i.e. time to follow-up colonoscopy and also known as monitoring time), 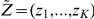 denote the *K* auxiliary variables, and *M* denote an indicator variable showing whether the follow-up colonoscopy was conducted at the end of the trial, i.e. I[*T ≥ τ*], where *τ* is the study duration, or equivalently the maximum follow-up. Suppose the study includes a random sample of *n* subjects. We use 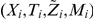 to denote the corresponding variables for the *i^th^* subject, *i = 1,…,n*. Note here for simplicity, we assume each participant has only one follow-up colonoscopy to mimic the structure of current status data. For each subject *I*, the observable data is ***O*** *= (T_i_, Δ_i_)*, where *T_i_≤τ* and *Δ_i_ = I*[*min(X_i_, T_i_) = X_i_*] is the recurrence indicator, and the observed data is denoted as *O = (t_i_, δ_i_)*. The time to recurrence for a participant *i* who had recurrence can be said to be interval censored into an interval [*0, t_i_*] with 0<*t_i_*<*τ*. The time to recurrence for a participant *j* who had no recurrence by the end of the trial can be said to be right censored at *t_j_*. Hsu et al. [1] showed that the Kaplan-Meier estimator derived from the right endpoint of the interval-censored data tends to overestimate the recurrence rate at the end of the trial under an assumption of independent censoring.

### 1. A Review of the Weighted Logistic Regression Method without Auxiliary Variables

The estimate of the recurrence rate based on the sample proportion can be expressed as 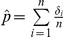. Assuming independent censoring, the expectation of 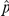 is equal to 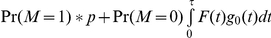, where *p* is the true recurrence rate at the end of the trial, *F*(.) is the distribution function of the time to recurrence *X*, and *g_0_*(.) is the conditional density function of the censoring time *T* given *T≤τ* (see the proof in Appendix S1). This implies that the estimate of the recurrence rate using the simple sample proportion without accounting for variable follow-up lengths will underestimate the recurrence rate and the bias is equal to 
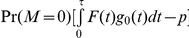
 under the assumption of independent censoring. The magnitude of the bias depends on the distributions of the times to recurrence and censoring, and the percentage of participants who had an early follow-up colonoscopy, i.e. Pr(*M = 0*). Clearly, the bias increases with the rate of having an early colonoscopy.

Hsu et al. [1] first proposed to directly estimate the recurrence rate at the end of the trial to avoid the problem associated with the possibility of cure in estimation. They then used a weight function *w(t)*, where *0≤w(t)≤1*, to incorporate censoring time into logistic regression for estimating the recurrence rate at the end of the trial. The weight function, an increasing function of the censoring time *t*, is used to represent the proportion of information captured regarding the recurrence status at the end of the trial for a participant. Specifically, *w(t) = 1*, if a participant was right or interval censored at the end of the trial, i.e. *t = τ*. In other words, the information regarding recurrence status at the end of the trial is fully available. Otherwise, the information is considered only partially available. It can be similarly seen that *w(t) = 1* if *δ = 1*. This approach assumes that a censored participant with a longer censoring (follow-up) time provides more information about the recurrence status at the end of the trial than a censored participant with a shorter censoring time. There are several ways to choose the weight function. In this paper, we only focus on a weight function derived from an exponential distribution, i.e. 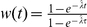, where 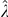 is the estimated hazard rate based on the observed recurrence time data [1]. Hsu et al. [1] showed that this approach produces a reasonable recurrence rate estimate under a variety of distributions for the time to recurrence even assuming a high early follow-up colonoscopy (or censoring) rate. This weight function is linearly incorporated into the logistic regression, i.e. the recurrence rate at time t is assumed to be w(t)*p. The resulting logistic regression is a weighted logistic regression (*wLogit*) model that adjusts for variable censoring time. The maximum likelihood estimator (MLE) of the recurrence rate, *p*, for the weighted logistic regression model is the solution of the equation 
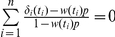
, which can be solved using the Newton-Raphson (NR) algorithm. The estimated variance of the MLE can be calculated using the Fisher information [1].

### 2. Weighted Logistic Regression Method with Auxiliary Variables

#### 2.1 Two Working PH Models

The auxiliary variables are assumed to be associated with the risk of recurrence and could be associated additionally with the risk of censoring. Hence, these variables can be incorporated into the weight function to adjust for variable censoring time and potential dependent censoring in estimating the recurrence rate at the end of the trial. However, directly incorporating the multiple auxiliary variables into the weight function requires nontrivial modeling which may introduce considerable computational burden into the estimation of the recurrence rate. We propose to simplify the modeling of the dependence of the weight function on the auxiliary variables through two steps. In the first step, we reduce the covariates to two risk scores. In the second step, we break the study population into different risk groups based on the categorized risk scores.

Specifically, in the first step we fit two working PH models to derive two risk scores to summarize the associations between the auxiliary variables and the recurrence and censoring times. One is a PH model with the auxiliary variables as the covariates for the recurrence time, where midpoint imputation is used to impute time to recurrence for those interval-censored participants who had recurrence before or at the end of the trial. The other is a PH model with the auxiliary variables as the covariates for the censoring time. The two risk scores are defined as 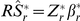 for the recurrence time model and 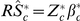 for the censoring time model, where 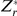 and 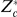 are the auxiliary variables included in the two working PH models, and 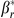 and 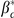 denote the estimates of the corresponding regression coefficients in the two working PH models, respectively. Note both 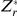 and 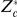 can be different from 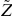. For example, 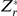 and 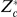 can include the interaction or transformed terms of the components of 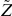.

Under the assumption that conditional on the auxiliary variables the recurrence time, *X*, is independent of the censoring time, *T*, we have the following result (with the proof given in Appendix S1): If one of the two working PH models is correct, X and T are asymptotically independent conditional on the two risk scores.

This result allows us to use the two risk scores to define risk groups for recurrence, within each of which the recurrence time is asymptotically approximately independent of the censoring time. This idea is analogous to propensity score matching [15]. Note that the risk groups are defined using the estimated risk scores since the true scores are unknown. The time to recurrence is either interval or right censored. Hence, the working PH model for the recurrence time based on the mid-point imputed data is considered misspecified even if the true recurrence time model is a PH model. The censoring time is always observed. The working PH model based on the observed censoring time data is considered correctly specified if the true censoring time model is a PH model with the specified auxiliary variables as covariates. This assumption on the censoring time model is the underlying assumption for our proposed estimation method, as will be described below.

#### 2.2 Estimation Scheme

We first categorize the two risk scores to define risk groups. These two risk scores can be categorized separately and be jointly used to define the risk groups. However, when there is dependent censoring of the recurrence time, these two risk scores could be highly correlated. This may result in few observations in some of the risk groups, making estimation unstable. To overcome this sparseness problem we propose using the principal component analysis on the two standardized (centered and scaled) risk scores to derive two orthogonal components (as linear combinations of the two risk scores) and then categorize these two components separately based on their percentiles into *g* ( = I * J) groups, where *I* is the number of categories for the first component and *J* is the number of categories for the second component. We describe next our proposed weighted logistic regression estimator based on the I*J categorized groups (denoted as *WLogit_I,J_*).

Given the assumption that the recurrence time is independent of the censoring time conditional on the auxiliary variables, within each risk group we can approximately assume that censoring is independent of the risk of recurrence. Therefore, the weighted logistic regression method [1], which relies on the independent censoring assumption, can be employed to estimate the group-specific recurrence rates. Let 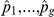 and 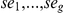 denote the recurrence rate estimates at the end of the trial and the associated standard error estimates derived from the weighted logistic regression models for the *g* risk groups, respectively. The final recurrence rate estimate and the associated standard error estimate can be expressed as 
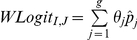
 and 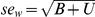, where 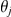 is the sample proportion of the participants in risk group *j*, 
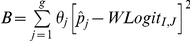
 (between group variation) and 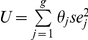 (within group variation). Note that the formula for *se_w_* is derived (with derivation omitted) using the fact that the estimates 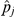 are independent across risk groups and assuming 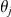 equals the true proportion of the participants in risk group *j*. The above procedures can be summarized into the following seven steps.

**Step 1.** Fit a PH model to the midpoint imputed recurrence time and the observed censoring time, respectively.

**Step 2.** Calculate the risk score for both PH models.

**Step 3.** Standardize the two risk scores, respectively, by subtracting the mean and dividing by the standard deviation.

**Step 4.** Perform principal component analysis on the two standardized risk scores to generate two orthogonal components.

**Step 5.** Categorize the two components to define risk groups.

**Step 6.** Perform the WLogit on the risk groups derived from Step 5 to obtain the group-specific recurrence rate.

**Step 7.** Calculate the final recurrence rate estimate and the associated standard error estimate using the group-specific estimates obtained in Step 6.

## Results

### 1. Application to the UDCA data

In 1996, the Arizona Cancer Center initiated a multi-center trial to assess whether ursodeoxycholic acid (UDCA) prevents the recurrence of colorectal adenomas. 1285 subjects with a history of colorectal adenomas were recruited and randomly assigned to one of the two treatment groups, placebo and UDCA (8–10 mg/kg/day) [16]. Out of the 1285 subjects, a total of 1192 subjects (placebo: 579; UDCA: 613) underwent at least one follow-up colonoscopy and were thus considered for the analysis of the recurrence status. The recurrent status of each participant was measured, as well as his/her baseline covariates. The baseline characteristics of the 1192 subjects were summarized in [16]. Among the 1192 subjects, 233 (40.2%) in the placebo group and 260 (42.4%) in the UDCA group had at least one early follow-up colonoscopy, 327 (27.4%) of which (across groups) had multiple follow-up colonoscopies. Instead of fixing the end of the trial exactly at three years, for each participant the actual time of the colonoscopy is used to define the interval of time to first recurrence. In other words, there is a different *τ*, say *τ_i_*, for each individual. The midpoint imputation method is then applied to the interval censored observations.

We explored the covariates associated with the risk of recurrence and censoring by fitting PH models to the midpoint-imputed recurrence data and the observed follow-up colonoscopy (censoring) time data, respectively. Based on those two PH models, we can first indentify the covariates associated with both recurrence and censoring to evaluate whether dependent censoring exists. We then include all of the covariates associated with both recurrence and censoring into the two working PH models to derive the risk groups to ensure that the dependence has been captured. The results, as summarized in Table 1, indicate that there appears to be auxiliary variables associated with the risks of both recurrence and censoring, and there is potential dependent censoring in the UDCA data. Specifically, for the placebo group, age, gender, polyp history, size of the largest baseline adenoma (≥1 cm or not) and multiplicity (number of baseline adenomas) are significantly associated with the risk of recurrence; the polyp history is marginally associated with the risk of censoring. This suggests potential dependent censoring in the placebo group although the magnitude might be weak. For the UDCA group, BMI (≥25 or not), size of the largest baseline adenoma and multiplicity are significantly associated with the risk of recurrence; family history of colorectal cancer and size are significantly associated with the risk of censoring in the UDCA group. The size of the largest adenoma is significantly associated with both risks of recurrence and censoring. These results suggest that there is potential dependent censoring in the UDCA group. In addition, a goodness of fit approach based on Schoenfeld residuals was used to verify the PH assumption for both midpoint-imputed time to recurrence and time to censoring.

**Table 1:** UDCA Study: Univariate analysis based on a PH model.

	Time to Recurrence					
	Placebo			UDCA		
Variable	HR	95% CI	p-value	HR	95% CI	p-value
Age	1.026	1.010, 1.042	<0.01	1.010	0.995, 1.025	0.20
BMI (≥25)	1.210	0.914, 1.602	0.18	1.464	1.087, 1.970	0.01
Male	1.312	1.002, 1.719	0.05	1.235	0.936, 1.630	0.14
Previous polyp history	1.378	1.070, 1.774	0.01	1.029	0.798, 1.327	0.82
Family history of CRC^a^	1.001	0.766, 1.310	0.99	1.024	0.774, 1.355	0.87
Size (≥1 cm)	1.418	1.108, 1.815	0.01	1.718	1.340, 2.203	<0.01
Multiplicity	1.678	1.311, 2.149	<0.01	1.921	1.498, 2.463	<0.01

	Time to Censoring					
	Placebo			UDCA		
Variable	HR	95% CI	p-value	HR	95% CI	p-value
Age	1.008	0.995, 1.020	0.23	1.004	0.992, 1.016	0.49
BMI (≥25)	1.207	0.949, 1.535	0.13	1.127	0.899, 1.413	0.30
Male	1.002	0.799, 1.256	0.99	0.999	0.802, 1.244	0.99
Previous polyp history	0.820	0.650, 1.035	0.09	0.849	0.687, 1.051	0.13
Family history of CRC^a^	0.944	0.742, 1.199	0.63	0.777	0.610, 0.989	0.04
Size (≥1 cm)	0.999	0.798, 1.252	1.00	1.303	1.051, 1.615	0.02
Multiplicity	0.960	0.756, 1.220	0.74	1.059	0.850, 1.319	0.61

a colorectal cancer.

Based on the above preliminary findings, for the placebo group we include the five significant covariates in fitting the working PH model for the midpoint-imputed recurrence time data to derive a risk score of recurrence. The risk score of recurrence is categorized into four groups by the quartiles and the dichotomous polyp history variable is directly used as the risk score of censoring. The weighted logistic regression estimation is then performed separately in each of the 4*2 groups (denoted as *WLogit_4,2_*). For the UDCA group, we include the three significant covariates in the working PH model for the midpoint-imputed recurrence time data to derive a risk score of recurrence. We include the two significant covariates in the working PH model for the observed censoring time data to derive a risk score of censoring. The two risk scores are standardized to perform principal component analysis to derive two orthogonal components. Note that we conduct principal component analysis for the UDCA group because of the potentially high correlation between the two risk scores in the UDCA group. These two principal components are then categorized separately into four and two categories, respectively, based on their percentiles. The weighted logistic regression estimation is then performed in the 4*2 groups (denoted as *WLogit_4,2_*). In addition, for both the placebo and UDCA groups we also perform the weighted logistic regression estimation without using the two risk scores (denoted as *WLogit*), using only the risk score of recurrence (denoted as *WLogit_4,1_*), where the risk score is categorized into four groups by the quartiles, and using only the risk score of censoring (denoted as *WLogit_1,2_*), where the risk score is dichotomized into two groups by the median. For all weighted logistic regression methods, the weights are derived from an exponential distribution truncated at three years.

In addition to the weighted logistic regression methods, we also calculate the sample proportion of recurrence (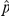) and the NPMLE. The results are provided in Table 2. The sample proportion method produces a lower recurrence rate estimate than the NPMLE and weighted logistic regression methods for both the placebo and UDCA groups. This is consistent with our previous findings [1]. All of the weighted logistic regression methods, especially for the WLogit method, produce higher recurrence rate estimates than 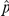 and the NPMLE in both the placebo and UDCA groups. The WLogit method that does not incorporate the two risk scores into estimation produces an odds ratio estimate of 0.869 (very close to the estimate of 0.887 based on the sample proportion) and a 95% confidence interval (CI) of (0.674, 1.157) for the treatment effect. All three weighted logistic regression methods incorporating the auxiliary variables, i.e. *WLogit_4,2_*, *WLogit_4,1_* and *WLogit_1,2_*, produce lower odds ratio estimates. Specifically, the *WLogit_4,2_* method produces an odds ratio estimate of 0.767 with a 95% CI of (0.601, 0.988), the *WLogit_4,1_* produces an odds ratio estimate of 0.763 with a 95% CI of (0.587, 0.988), and the WLogit_1,2_ method produces an odds ratio estimate of 0.752 with a 95% CI of (0.590, 0.981). None of the 95% CIs include one, suggesting that UDCA is associated with a lower risk of recurrence. In contrast, none of the sample proportion, NPMLE and WLogit methods finds this significant treatment difference. Of the three methods, *WLogit_1,2_*, which only incorporates the risk score from the censoring time into the analysis, produces the lowest estimate of the odds ratio. In summary, the proposed extended weighted logistic regression method provides an adjustment for dependent censoring by using information from the auxiliary variables.

**Table 2:** UDCA Study: Estimation of recurrence rate for placebo and UDCA groups at 3 years.

	Placebo		UDCA		
Method	estimate	SE^a^	estimate	SE	OR (95% CI^b^)
NPMLE^c^	0.467	0.031	0.414	0.032	0.807 (0.545, 1.136)
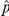	0.439	0.020	0.409	0.020	0.887 (0.705, 1.122)
WLogit	0.539	0.023	0.504	0.023	0.869 (0.674, 1.157)
WLogit_4,2_	0.525	0.024	0.459	0.021	0.767 (0.601, 0.988)
WLogit_4,1_	0.525	0.024	0.457	0.021	0.763 (0.587, 0.988)
WLogit_1,2_	0.526	0.025	0.455	0.021	0.752 (0.590, 0.981)

a standard error.

b derived from 500 bootstrap samples.

c estimate of the standard error derived from 500 bootstrap samples.

### 2. Simulation study

We perform a simulation study to investigate the small sample performance of the proposed methods for estimating the recurrence rate in the presence of auxiliary variables. We focus on the comparison among the NPMLE, sample proportion (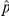) and the three weighted logistic regression methods (*WLogit*, *WLogit_4,1_*, *WLogit_4,2_*). In each of 1000 simulated data sets, there are five hypothetical auxiliary variables (z_1_,…,z_5_) independently generated from a *Uniform(0,1)* distribution. Each participant's recurrence time (*X*) is generated from a hypothetical PH model conditional on the five auxiliary variables, where the hazard function is 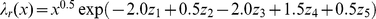. For simplicity, we assume every participant only has one follow-up colonoscopy. The follow-up colonoscopy (censoring) time is generated from a hypothetical distribution truncated at three years (i.e. the end of the trial), in which the parameters of the distribution are selected to constrain the probability of having an early colonoscopy in a pre-specified range of 30∼50%. In the case of independent censoring, the time to follow-up colonoscopy (*T*) is generated from an exponential distribution with a constant hazard 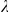 and truncated at three years. In the case of dependent censoring, the time to follow-up colonoscopy is generated from a hypothetical PH model conditional on the auxiliary variables with a hazard function 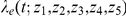 and truncated at three years. For a participant who has the follow-up colonoscopy at three years, the recurrence time is then either censored in the interval [0, 3] or right censored at three years. For a participant who has the follow-up colonoscopy before three years, the recurrence time is then either censored in the interval (0, *t_i_*] or right censored at *t_i_*. In order to fit the two working PH models, each participant is considered either right censored at *t_i_* or three years or having recurrence at the midpoint of the interval (0, *t_i_*] or (0, 3] (i.e. *t_i_*/2 or 1.5). A working PH model with the five hypothetical auxiliary variables as the covariates is fitted to the midpoint-imputed recurrence time data and the observed censoring time data, respectively, to derive two risk scores. The principal component analysis is then conducted on the two standardized (centered and scaled) risk scores to derive two orthogonal components. These two components are used to define the risk groups for performing the weighted logistic regression estimation. Specifically, for the *WLogit_4,1_* method only the first orthogonal component is used to define four risk groups based on its quartiles. For the *WLogit_4,2_* method both orthogonal components are used to define eight groups, where the first component is categorized into four groups based on its quartiles and the second component is dichotomized into two groups based on its median. A sample size of 200 and various rates of having early colonoscopy are considered. The standard error estimate for each of the 1000 datasets using the NPMLE method is derived from 500 bootstrap samples.

The results are summarized in Tables 3 and 4. When censoring is independent of the risk of recurrence (Table 3), as expected, the sample proportion 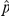 has the largest bias in estimating the recurrence rate at three years (the end of the trial) compared to the other methods in all scenarios. The bias also results in a low coverage rate for the sample proportion method. The NPMLE and weighted logistic regression methods all produce a point estimate close to the true recurrence rate and a coverage rate close to the nominal level. As the rate of having early follow-up colonoscopy increases, the bias slightly increases for the NPMLE, *WLogit_4,1_* and *WLogit_4,2_* methods. The bias results in a coverage rate slightly off from the nominal level for the NPMLE, *WLogit_4,1_* and *WLogit_4,2_* methods when the rate of having early follow-up colonoscopy is as high as approximately 50%. The three weighted logistic regression methods yield similar point estimates and coverage rates. The weighted logistic regression methods gain efficiency ranging from 10% to 23% in terms of the empirical variance compared to the NPMLE method. The weighted logistic regression methods gain efficiency mainly through adjustment of the variable follow-up lengths, instead of by incorporating the auxiliary variables into the weight function.

**Table 3:** Monte Carlo Results: Estimation of recurrence of adenomas at 3 years (true recurrence rate: 0.495) assuming independent censoring with 5 auxiliary variables.

Method	Est^a^	Bias	SD^b^	SE^c^	CR^d^
λ^e^ = 0.12; 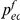 [Table-fn nt110] = 0.30; early censoring rate^g^ = 22.8%; |r^h^| = 0.37					
NPMLE	0.500	0.005	0.0401	0.407	94.4
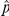	0.417	−0.078	0.0341	0.0348	36.3
WLogit	0.490	−0.005	0.0378	0.0390	95.0
WLogit_4,1_	0.490	−0.005	0.0385	0.0382	94.7
WLogit_4,2_	0.490	−0.005	0.0380	0.0379	94.6
λ = 0.17; p_ec_ = 0.40; early censoring rate = 30.7%; |r| = 0.37					
NPMLE	0.503	0.008	0.0435	0.0433	94.2
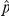	0.390	−0.105	0.0340	0.0344	13.4
WLogit	0.490	−0.005	0.0398	0.0407	95.5
WLogit_4,1_	0.490	−0.005	0.0411	0.0396	93.2
WLogit_4,2_	0.489	−0.006	0.0404	0.0391	94.0
λ = 0.23; p_ec_ = 0.50; early censoring rate = 38.7%; |r| = 0.36					
NPMLE	0.508	0.013	0.0481	0.0468	93.0
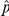	0.361	−0.134	0.0341	0.0339	2.5
WLogit	0.490	−0.005	0.0428	0.0427	94.3
WLogit_4,1_	0.488	−0.007	0.0432	0.0411	93.2
WLogit_4,2_	0.488	−0.007	0.0421	0.0406	93.9

a average of 1000 estimated recurrence rates;

b empirical standard deviation of 1000 point estimates.

c average of 1000 estimated standard errors;

d fraction of 95% CIs which contains the true value.

e hazard rate for time to colonoscopy;

f proportion of participants with early colonoscopy.

g right censoring occurs before 3 years;

h Spearman correlation coefficient between the 2 risk scores.

**Table 4:** Monte Carlo Results: Estimation of recurrence of adenomas at 3 years (true recurrence rate: 0.495) assuming dependent censoring with 5 auxiliary variables.

Method	Est	Bias	SD	SE	CR
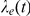 a 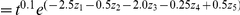 ; p_ec_ = 0.30; early censoring rate = 19.9%; |r| = 0.83					
NPMLE	0.448	−0.047	0.0386	0.0388	76.4
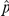	0.398	−0.097	0.0341	0.0345	18.6
WLogit	0.460	−0.035	0.0378	0.0384	85.1
WLogit_4,1_	0.500	0.005	0.0397	0.0381	93.6
WLogit_4,2_	0.492	−0.003	0.0382	0.0382	94.6
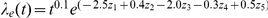 ; p_ec_ = 0.40; early censoring rate = 27.7%; |r| = 0.86					
NPMLE	0.418	−0.077	0.0403	0.0403	52.5
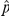	0.356	−0.139	0.0347	0.0337	2.4
WLogit	0.438	−0.057	0.0400	0.0396	70.2
WLogit_4,1_	0.489	−0.006	0.0412	0.0399	93.4
WLogit_4,2_	0.482	−0.013	0.0405	0.0401	93.1
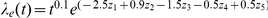 ; p_ec_ = 0.50; early censoring rate = 36.8%; |r| = 0.79					
NPMLE	0.409	−0.086	0.0425	0.0431	47.2
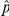	0.322	−0.173	0.0333	0.0329	0.2
WLogit	0.432	−0.063	0.0403	0.0416	67.2
WLogit_4,1_	0.480	−0.015	0.0426	0.0431	93.8
WLogit_4,2_	0.488	−0.007	0.0421	0.0406	93.9

a hazard function for time to follow-up colonoscopy.

In the case where dependent censoring is present (Table 4), as expected, the NPMLE, sample proportion and *WLogit* methods all produce biased estimates of the recurrence rate, with the largest biases being associated with the sample proportion method. The bias for all three methods increases with the rate of having an early follow-up colonoscopy. The biases similarly result in low coverage rates of the 95% CIs, especially for the sample proportion method. The NPMLE and *WLogit* methods are biased because they assume that censoring is independent of the risk of recurrence. The two proposed weighted logistic regression methods, *WLogit_4,1_* and *WLogit_4,2_*, which incorporate the auxiliary variables into the weight function produce point estimates comparable to the true recurrence rate and the coverage rates close to the nominal level in all scenarios. When the rate of having an early follow-up colonoscopy increases, we do observe the bias slightly increases for the two methods, especially for the *WLogit_4,2_* method. This suggests that incorporating the second component derived from the principal component analysis does not improve the estimation of the recurrence rate.

In summary, the results in Tables 3 and 4 show that the sample proportion method tends to produce biased estimates of the recurrence rate at the end of the trial in both cases of independent and dependent censoring. The NPMLE and *WLogit* methods both can produce reasonable estimates in the case of independent censoring yet biased estimates in the case of dependent censoring. The weighted logistic regression methods that adjust for the potential dependent censoring using information from the auxiliary variables gain efficiency compared to the NPMLE method and reduce bias due to dependent censoring compared to the NPMLE and *WLogit* methods.

## Discussion

In this paper we propose an extended weighted logistic regression method for estimating the rate of recurrence of adenomas in the presence of multiple auxiliary variables predictive of the recurrence and potential dependent censoring. The auxiliary variables are summarized into two risk scores by fitting two working PH models. The two risk scores are then categorized to define risk groups. This approach is based on the assumption that the auxiliary variables capture all the dependence between recurrence and censoring times. Our simulation results show that the weighted logistic regression methods incorporating the auxiliary variables into the weight function improve efficiency and reduce bias due to dependent censoring in the estimation of the recurrence rate. In contrast, the sample proportion of recurrence tends to underestimate the recurrence rate, and the NPMLE and WLogit methods, which all rely heavily on the assumption of independent censoring, produce biased estimates when censoring is dependent upon the risk of recurrence. Hence, the methods that do not account for variable censoring time or dependent censoring for estimating the recurrence rate could produce misleading results as indicated in the analysis of the UDCA data. In addition, the weighted logistic regression approach can also easily accommodate covariates in the model. Although the proposed method in this paper is mainly motivated by data from colorectal polyp prevention trials, the method can be also used to handle potential dependent censoring for cancer research with a similar design, for example, study of Human Papillomavirus (HPV) reinfection where each participant comes in to have the HPV test every 3 or 6 months and might dropout from the study due to the reasons related to risk of HPV.

As we point out earlier, the weight will only affect the contribution of the participants who are censored before the end of the trial toward estimating the recurrence rate at the end of the study. The main endpoint of a typical polyp prevention trial is often the recurrence rate at the end of the trial. Because often a large proportion of (e.g., 50%), participants have their only follow-up colonoscopy at the end of the trial, the proposed approach is considered to be more stable and less dependent on the two working PH models than the survival techniques. The working PH model for the recurrence time is based on the midpoint-imputed data. The midpoint imputation has been shown to be associated with bias in survival analysis [17]. However, the proposed weighted logistic regression method that only uses midpoint imputation to derive a risk score of recurrence has been shown to be less affected by bias and more efficient than the NPMLE method in our simulation study. The censoring times before or at the end of the trial are always observed. Hence, of the two working PH models, one should focus on correctly specifying the working PH model for the censoring time.

One might feel that more sophisticated (and therefore usually computationally intensive) approaches for fitting the two working PH models should be used. However, we suspect that this type of approach would not lead to a significant reduction in bias, which is the major concern in the case of dependent censoring for the original weighted logistic regression method [1]. In addition, parametric assumptions in the PH models are only employed to define the risk scores. As a result, the reliance on the correct specification of the PH models is weaker for the modified weighted logistic regression method. In the estimation of the recurrence rate in the presence of dependent censoring, the improvement in efficiency by the weighted logistic regression method using predictive covariates of recurrence will still depend on the strength of the association between the auxiliary variables and recurrence.

In this paper, we only consider several combinations of *I* and *J* for the number of risk groups. The performance of the weighted logistic regression approach depends on the “closeness” of the risk groups. The closeness of the risk groups mainly depends on how many risk groups are defined by categorizing the two risk scores. One would expect that as *I* and/or *J* increases, each individual risk group becomes more likely to be homogeneous, but this could increase the chance of having an unstable recurrence rate estimate for all risk groups due to the reduced sample size for each risk group. Hence, the total number of risk groups to be categorized depends on the sample size. In practice, one may want to assure that each risk group has sufficient number of observations, say 20, to obtain a stable estimate of the weight function. As for how to choose the ratio between *I* and *J*, we recommend choosing it by calculating the percentage of variance in the two standardized risk scores explained by each component. For example, if the first and second components each account for 2/3 and 1/3 of the total variance in the two standardized risk scores, respectively, we might want to choose a ratio of *I/J* of 2.

## Supporting Information

Appendix S1 We first derive the bias for the sample proportion estimate and then establish the property of the two working models, which are used to derive the two risk scores to define risk groups for recurrence. The property indicates that within each of the risk groups the recurrence time is asymptotically approximately independent of the censoring time.
(DOC)
