# Supplementary material for: Estimation of Recurrence of Colorectal Adenomas with Dependent Censoring Using Weighted Logistic Regression
Source: PLoS One. 2011 Oct 31;6(10):e25141. doi: 10.1371/journal.pone.0025141 (PMC3204965; doi:10.1371/journal.pone.0025141)
Supplement: Appendix S1 — We first derive the bias for the sample proportion estimate and then establish the property of the two working models, which are used to derive the two risk scores to define risk groups for recurrence. The property indicates that within each of the risk groups the recurrence time is asymptotically approximately independent of the censoring time. (DOC) [file pone.0025141.s001.doc]

**Appendix S1**

*A.1. Derivation of the bias for the sample proportion estimate:*

$E(\hat{p})=\sum_{i=1}^{n} \frac{\delta_{i}(t_{i})}{n}$and$E(\delta(t))=\Pr(M=1)E[\delta(t)|M=1]+\Pr(M=0)E[\delta(t)|M=0)$, which is equal to $\Pr(M=1)p+\Pr(M=0)\int_{0}^{\tau} F(t)g_{0}(t)dt$, where *p* is the true recurrence rate at the end of the trial, *F*(.) is the distribution function of the time to recurrence *X*, and *g_0_*(.) is the conditional density function of the follow-up time *T* given *T* ≤ τ. Hence, $E(\hat{p})$ is less than *p* because *F*(t) ≤ *p* for all *t* ≤ τ.

*A.2. Proof of Result 1*: We only consider the situation where the working recurrence time model is incorrect and the working follow-up colonoscopy time model is correct, i.e. $\Pr(T>s|Z)=\Pr(T>s|\beta_{c}Z)$, where $\beta_{c}$ and $Z$ are the regression coefficients and covariates, respectively, for both the true and working PH follow-up colonoscopy time model, and $Z_{c}^{*}=Z$and$\beta_{c}^{*}=\beta_{c}$. Hence, the follow-up colonoscopy time *T* depends on Z only through $\beta_{c}Z$. Let *RS*^*^ denote the vector of the two risk scores from the two working PH models with $\beta_{r}^{*}$ and $\beta_{c}^{*}(\text{ or }\beta_{c})$ assumed known. Note that despite the misspecification of the working PH model for the recurrence time, based on [14] under appropriate regularity conditions there always exists a constant vector $\beta_{r}^{*}$ that is the limit (as the mean in the root-*n* asymptotic normal distribution) of the estimated coefficients from the (misspecified) working PH model for the recurrence time, as the sample size goes to infinity. In other words, $\beta_{r}^{*}$ is well defined. Clearly, $\beta_{c}^{*}$ is also well defined because the working PH model for the censoring time is the true model. Thus we have

$$\Pr(X>s_{1},T>s_{2}|RS^{*})=E_{Z|RS^{*}}[E\{I(X>s_{1},T>s_{2})|Z,RS^{*}\}]=E_{Z|RS^{*}}[\Pr(X>s_{1}|Z)\Pr(T>s_{2}|Z)]$$

$$\begin{matrix} =E_{Z|RS^{*}}[\Pr(X>s_{1}|Z)\Pr(T>s_{2}|\beta_{c}^{*}Z_{c}^{*})]=E_{Z|RS^{*}}[\Pr(X>s_{1}|Z)]\Pr(T>s_{2}|RS^{*}) \\ =\Pr(X>s_{1}|RS^{*})\Pr(T>s_{2}|RS^{*}). \end{matrix}$$

The second equality holds because of the assumption of conditional independence between the times of recurrence and follow-up colonoscopy given *Z*. The third equality holds under the assumption that the working follow-up colonoscopy time model is correctly specified. Again, under appropriate regularity conditions, the estimates of the regression coefficients from both the working recurrence and censoring time models converge (in the sense of root-*n* asymptotic normality) to $\beta_{r}^{*}$ and $\beta_{c}^{*}(\text{ or }\beta_{c})$, respectively, as the sample size goes to infinity. The asymptotic independence between *X* and *T* then follows.
